# Central Suppression of the GH/IGF Axis and Abrogation of Exercise-Related mTORC1/2 Activation in the Muscle of Phenotype-Selected Male Marathon Mice (DUhTP)

**DOI:** 10.3390/cells10123418

**Published:** 2021-12-04

**Authors:** Julia Brenmoehl, Christina Walz, Caroline Caffier, Elli Brosig, Michael Walz, Daniela Ohde, Nares Trakooljul, Martina Langhammer, Siriluck Ponsuksili, Klaus Wimmers, Uwe K. Zettl, Andreas Hoeflich

**Affiliations:** 1 Institute for Genome Biology, Research Institute for Farm Animal Biology (FBN), Wilhelm-Stahl-Allee 2, 18196 Dummerstorf, Germany; brenmoehl@fbn-dummerstorf.de (J.B.); walz@fbn-dummerstorf.de (C.W.); caro.caffier@yahoo.de (C.C.); e.brosig@web.de (E.B.); walz.michael@fbn-dummerstorf.de (M.W.); ohde@fbn-dummerstorf.de (D.O.); trakooljul@fbn-dummerstorf.de (N.T.); ponsuksili@fbn-dummerstorf.de (S.P.); wimmers@fbn-dummerstorf.de (K.W.); 2 Department of Neurology, Neuroimmunological Section, University Medicine Rostock, Gehlsheimer Str. 20, 18147 Rostock, Germany; uwe.zettl@med.uni-rostock.de; 3 Lab Animal Facility, Research Institute for Genetics and Biometry, Institute for Farm Animal Biology (FBN), Wilhelm-Stahl-Allee 2, 18196 Dummerstorf, Germany; martina.langhammer@fbn-dummerstorf.de

**Keywords:** endurance exercise, energy metabolism, pituitary gland, muscle, growth hormone, insulin-like growth factor, mTORC, PTEN, Ragulator complex, sirtuins, mouse model

## Abstract

The somatotropic axis is required for a number of biological processes, including growth, metabolism, and aging. Due to its central effects on growth and metabolism and with respect to its positive effects on muscle mass, regulation of the GH/IGF-system during endurance exercise is of particular interest. In order to study the control of gene expression and adaptation related to physical performance, we used a non-inbred mouse model, phenotype-selected for high running performance (DUhTP). Gene expression of the GH/IGF-system and related signaling cascades were studied in the pituitary gland and muscle of sedentary males of marathon and unselected control mice. In addition, the effects of three weeks of endurance exercise were assessed in both genetic groups. In pituitary glands from DUhTP mice, reduced expression of Pou1f1 (*p* = 0.002) was accompanied by non-significant reductions of Gh mRNA (*p* = 0.066). In addition, mRNA expression of Ghsr and Sstr2 were significantly reduced in the pituitary glands from DUhTP mice (*p* ≤ 0.05). Central downregulation of Pou1f1 expression was accompanied by reduced serum concentrations of IGF1 and coordinated downregulation of multiple GH/IGF-signaling compounds in muscle (e.g., Ghr, Igf1, Igf1r, Igf2r, Irs1, Irs2, Akt3, Gskb, Pik3ca/b/a2, Pten, Rictor, Rptor, Tsc1, Mtor; *p* ≤ 0.05). In response to exercise, the expression of Igfbp3, Igfbp 4, and Igfbp 6 and Stc2 mRNA was increased in the muscle of DUhTP mice (*p* ≤ 0.05). Training-induced specific activation of AKT, S6K, and p38 MAPK was found in muscles from control mice but not in DUhTP mice (*p* ≤ 0.05), indicating a lack of mTORC1 and mTORC2 activation in marathon mice in response to physical exercise. While hormone-dependent mTORC1 and mTORC2 pathways in marathon mice were repressed, robust increases of Ragulator complex compounds (*p* ≤ 0.001) and elevated sirtuin 2 to 6 mRNA expression were observed in the DUhTP marathon mouse model (*p* ≤ 0.05). Activation of AMPK was not observed under the experimental conditions of the present study. Our results describe coordinated downregulation of the somatotropic pathway in long-term selected marathon mice (DUhTP), possibly via the pituitary gland and muscle interaction. Our results, for the first time, demonstrate that GH/IGF effects are repressed in a context of superior running performance in mice.

## 1. Introduction

As a neuroendocrine organ in the brain, the pituitary gland acts as a mediator of central signals to peripheral tissues. It is required for normal growth and development but also has adaptive functions. Already in 1963, it was demonstrated that the secretion of growth hormone (GH) could be induced by physical exercise [1]. However, the mechanisms and conditions related to GH secretion under resistance and aerobic exercise conditions are still debated [2]. Elevated secretion of GH in response to resistance exercise can be seen in the context of hypertrophic muscle growth, and the misuse of GH as an agent for muscle accretion has a long but inglorious tradition [3]. However, the role of GH in response to endurance exercise is not directly evident because endurance exercise is less a direct function of muscle mass but more related to energy-metabolic adaptation in muscle but also in the liver and fat depots.

Inside the cell, hormonal stimuli are mediated by signaling cascades, such as PI3K, controlling mTOR activity via S6K [4]. And although the direct connection between GH/IGF and hypertrophic muscle growth is plausible in general, direct activation of IGF-depending PI3K/mTOR/S6K by exercise so far cannot be proven. In addition, although a number of studies have demonstrated GH-secretion in response to physical activity, so far, elevated activity of free IGF1 in serum cannot be proven [5]. In fact, there is experimental evidence that mTOR/S6K-dependent hypertrophic muscle growth is induced by IGF-independent mechanisms [6,7], raising the question of the molecular function of the GH/IGF system in the adaptive response to physical exercise.

Concerning the fundamental question about the role of the GH/IGF axis in physical exercise, we asked if and how the somatotrophic axis is regulated in born marathon runners. We addressed this question in a mouse model (DUhTP) selected for more than 140 generations of high-endurance exercise on a treadmill. DUhTP mice acquired running capacities three to four times higher than unselected controls (DUC mice) during the selection experiment [8]. Importantly, DUhTP mice were kept in the absence of running wheels in their home cages; accordingly, the running capacities are based on genetic adaptation but not on self-training. Moreover, the selection experiment was performed starting from an outbred background while avoiding inbreeding during selection; therefore, we assume multiple genetic adaptations in response to the selection pressure in DUhTP mice.

In the present study, we compared marathon mice and unselected controls, but in addition, we investigated the effects of three weeks of endurance exercise on the somatotropic axis in both mouse lines. To do so, we compared a genetic model (DUhTP vs. DUC mice) and an experimental model (sedentary versus trained). We used two related RNAseq experiments, which studied mRNA expression in the pituitary gland [9] and muscle (Brenmoehl et al., submitted) by integrating the genetic model and training effects. Here, for the first time, we considered the interaction of two different tissues, the pituitary gland and muscle, with respect to phenotype selection and training. Finally, hypotheses derived from the interpretation of mRNA expression were tested on the protein level by analyzing intra-cellular signal transduction.

## 2. Materials and Methods

### 2.1. Animals, Study Design, and Sample Preparation

The present study is based on an animal experiment described previously [9]. All experiments adhered to national and international standards, guidelines, and laws and were approved by the internal institutional committee and the state of Mecklenburg-Vorpommern (State Office for Agriculture, Food Safety, and Fisheries; AZ 7221.3-1-014/17, date of approval: 25 April 2017 and AZ 7221.3-1-064/19, date of approval: 30 January 2020). Long-term selected mice of the Dummerstorf high treadmill line (DUhTP) were compared with their corresponding unselected control (DUC) without (sed) and with three weeks of treadmill training adapted to the performance capacity of the respective line (trained). According to the three-week training program, the mice were running five days per week (Monday to Friday) starting at the age of 49 days after birth [9]. All mice were sacrificed at day 70 of life, and different tissues were collected, including the pituitary gland and *Musculus rectus femoris* (Mrf). Furthermore, serum and plasma samples were produced from fresh blood samples. For the generation of serum, the samples were centrifuged for 10 min at 1500× *g* at room temperature after incubation at room temperature for 30 min, and the supernatants were transferred to fresh 1.5 mL vials and finally centrifuged for 5 min at 1500× *g* at room temperature. The supernatants were then stored in fresh 1.5 mL vials at −20 °C. For plasma production, fresh blood was collected in a 1.5 mL vial containing 5 µL 500 mM ethylenediaminetetraacetic acid (EDTA). The samples were mixed and centrifuged at 5000× *g* for 10 min at 8 °C before the supernatants were stored at −20 °C.

### 2.2. Next-Generation Sequencing (NGS), Differential Gene Expression Analysis, and Data Processing

Isolation of total RNA derived from pituitary glands and Mrf, the generation of the DNA-Library, and the NGS procedure were performed and validated as previously described [9,Brenmoehl et al., submitted]. The obtained data were analyzed by comparing gene expression in the genetic groups (DUhTP and DUC) as well as the treatment groups (sedentary and trained). The following comparisons were made: DUhTP sed vs. DUC sed, DUhTP trained vs. DUC trained, DUC trained vs. DUC sed, and DUhTP trained vs. DUhTP sed. The effects were expressed as logarithmic fold change (log_2_ FC) with associated false discovery rate (FDR) [9]. As significantly regulated, a threshold FDR of 0.1 was set, and accordingly, differentially expressed genes (DEGs) were marked in red for higher expression and green for lower expression. To visualize more stringent regulation (FDR ≤ 0.05), the significant effects on the levels of gene expression were marked in bold.

### 2.3. IGF1 Assay

IGF1 concentrations in mouse sera and muscle lysates were determined using commercial ELISA kits (Mediagnost, Reutlingen, Germany) for mouse/rat IGF1 according to the manufacturer’s instructions. Serum was diluted at 1:100 with sample buffer (Mediagnost) before the determination. The Mrf tissue (≈50 mg) was combined with 10× (*w*/*v*) TE buffer (100 mM tris(hydroxymethyl)aminomethane, 10 mM EDTA, set to pH 7.5–8.0 with sodium dodecyl sulfate) and mechanically homogenized (6000 rpm, 2 min) in a vial with ceramic beads in the Precellys^®^24 (Peqlab Biotechnologie, Erlangen, Germany) and centrifuged (21,000× *g*) for 2 min at 4 °C after cooling on ice. The supernatant was diluted 1:2 with sample buffer and used in ELISA according to the manufacturer’s instructions. The concentration of IGF1 in muscle lysate is expressed concerning total protein concentration, determined by the bicinchoninic acid method (BCA Test Kit, SERVA Electrophoresis GmbH, Heidelberg, Germany).

### 2.4. Protein Isolation and SDS-PAGE

Again, ≈50 mg of muscle tissue were mechanically homogenized (6000 rpm for 2 × 30 s) in 1 × CST Cell Lysis Buffer (Cell Signaling, Frankfurt am Main, Germany) using Precellys ceramic beads. Samples were incubated on ice for 20 min and diluted 1:2 with 2× Laemmli (31.25 mM tris(hydroxymethyl)aminomethane, 1% sodium dodecyl sulfate, 5% glycerol). After denaturation (10 min, 94 °C) and centrifugation (2 min, 21,000× *g*, 4 °C), the protein concentration was also determined using the bicinchoninic acid method described earlier. The protein concentrations were then adjusted to 1 mg/mL with 1 × Laemmli, and beta-mercaptoethanol was added (to 0.4% finally). The samples (20 µg total protein) were separated by SDS-PAGE (Bio-Rad TGX Stain-Free FastCast Acrylamide kit; Bio-Rad Laboratories GmbH, Munich, Germany). After electrophoresis, proteins were transferred to a polyvinylidene fluoride (PVDF) membrane by semi-dry blotting (60 min, current 80 mA/gel).

### 2.5. Analysis of Signal Transduction by Western Immunoblotting (WIB)

For WIB, membranes were incubated in 3% dry milk in TBST (Tris-buffered saline with Tween20) for 1 h to block free binding sites of the membrane. After three washing steps, the membranes were incubated overnight at 4 °C with primary antibodies purchased from Cell Signaling Technology (CST, Danvers, MA, USA). Antibodies detecting total S6K (CST: #2708S) or phosphorylated S6K (CST: #9234S) were used at a dilution of 1:1000 in 3% bovine serum albumin (BSA). After five washing steps in TBST, the membranes were incubated for 2 h with the secondary antibody (anti-rabbit IgG HRP, #7074, dilution 1:2000, CST). The bands were visualized using Lumigen ECL Ultra (Lumigen Inc., Southfield, MI, USA) under a Bio-Rad station (Bio-Rad Chemi-Doc MP System, Bio-Rad Laboratories GmbH, Hercules, USA) with UV light and appropriate instrument software (Image Lab Ver. 6.0.1, Bio-Rad). In the relative calculation, the protein quantity of the samples was normalized to the total protein quantity.

### 2.6. Analysis of Signal Transduction by Capillary Immuno-Electrophoresis (WES^TM^)

For WES^TM^-analyses, protein samples were mixed with Fluorescent Master Mix (Protein Simple, San Jose, USA) and denatured for 5 min at 94 °C. The signal transduction analysis was performed with the WES^TM^ device from Protein Simple (San Jose, CA, USA) according to the manufacturer’s manual and as described before [10]. To perform the analysis, the following components were used: WES separation kit for 12–230 kDa with 8 × 25 capillary cartridges (#SM-W004-1), the affiliated standard pack (#PS-ST05-8), and an anti-rabbit detection module (DM-001). All devices and chemicals were purchased from Protein Simple. The analysis was performed with the software package Compass for Simple Western (Protein Simple). For the individual proteins, the following antibodies from CST were used (Danvers, Massachusetts, USA) with specific dilutions: Akt (CST: #9272; 1:50), phosphorylated Akt (CST: #9271; 1:20), p38 MAPK (CST: #9212; 1:50), phosphorylated p38 MAPK (CST: #4511; 1:20), PTEN (CST: #9188; 1:50), phosphorylated PTEN (CST: #9551; 1:3), and phosphorylated AMPKa (CST: #2535; 1:50). For AMPKa1/2 detection, the antibody sc-25792 from Santa Cruz Biotechnology (Dallas, TX, USA) was used with a dilution of 1:10. For analyses of phosphorylated PTEN, AMPKa1/2, and phosphorylated AMPKa, a sample dilution of 2 mg/mL and for the other analyses of 1 mg/mL were used according to the manufacturer’s protocol.

### 2.7. Statistical Analysis

All statistical analyses were performed using GraphPad Prism (version 9.0; GraphPad Software, San Diego, CA, USA). A linear balance line of known protein standard concentrations was used to calculate the measured protein concentrations. ANOVA for mixed models was used for significance testing, and pairwise comparisons were performed using the Tukey-Kramer method.

## 3. Results

### 3.1. Effects of Phenotype Selection and Running on the GH/IGF Axis in the Pituitary Gland and Muscle Tissue

To investigate effects of phenotype selection and treadmill running on somatic growth control and signal transduction, expression of respective candidate genes was assessed in the pituitary gland and muscle (Mrf) from marathon mice (DUhTP) and controls (DUC, Table 1).

**Figure 1:**
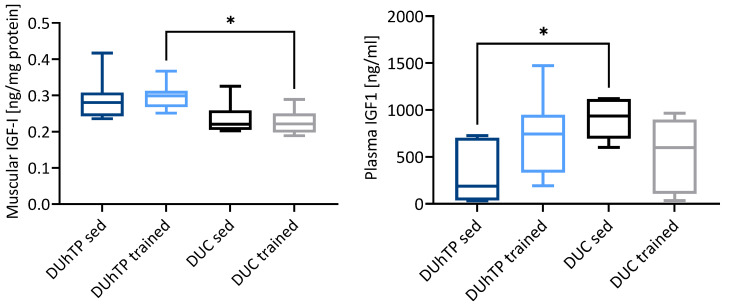
Effects of phenotype selection and three-week endurance exercise training on the concentrations of IGF1 in lysates from *Musculus rectus femoris* (**left**) and plasma (**right**). The concentrations of IGF1 in both matrices were determined by ELISA as described in the Materials and Methods. Results are presented as box plots. Statistical analysis was performed using one-way ANOVA. Significant differences are marked with an asterisk (* *p* < 0.05). Abbreviations are defined in Table 1.

In the pituitary gland of sedentary DUhTP mice, gene expression of the transcription factor for GH (Pou1f1, also known as Pit1), GH secretagogue receptor (Ghsr), Igf2, Igfbp2, and Pappa2 was reduced compared to sedentary DUC mice (*p* ≤ 0.05). Interestingly, gene expression of Pou1f1 and Igf2 was also reduced in DUC mice in response to exercise (*p* ≤ 0.05). In the pituitary gland, gene expression of growth hormone was reduced in sedentary DUhTP mice compared to sedentary DUC mice only with borderline significance (*p* = 0.066), whereas in trained DUhTP mice, the reduction of Gh mRNA expression was highly significant (*p* ≤ 0.001). In pituitary glands, expression of growth hormone-releasing hormone receptor (Ghrhr) mRNA was significantly increased in response to training in DUhTP versus DUC mice (*p* ≤ 0.005). In plasma samples from sedentary DUhTP mice, reduced concentrations of IGF1 were found compared to DUC mice (Figure 1; *p* ≤ 0.05).

In the femoral skeletal muscle, several members of the GH/IGF-system were affected in terms of the level of mRNA expression (Table 1). Notably, in addition to Igf1, mRNA expression for several receptors from the GH/IGF-system (Ghr, Igf1r, Igf2r) was reduced in both experimental DUhTP groups (*p* ≤ 0.05). In addition, the insulin receptor (Insr) and insulin receptor substrate 1 (Irs1) were reduced in both experimental DUhTP groups compared to their unselected control groups (*p* < 0.001). Instead, the expression of several IGFBPs was elevated in trained DUhTP mice (*p* ≤ 0.01). Contrasting the significantly reduced expression of IGF1 mRNA in muscle, protein levels of IGF1 in muscle tissue were higher in trained DUhTP mice compared to trained DUC mice (*p* ≤ 0.05; Figure 1).

### 3.2. Effects of Phenotype Selection and Endurance Exercise on mRNA Expression of Intracellular Signaling Compounds in the Pituitary Gland and Muscle

Hormonal signals from the pituitary gland can regulate intracellular signal transduction by autocrine or endocrine mechanisms. On the level of GH, IGFs, IGF-receptors, and IGFBPs, mainly inhibitory effects of phenotype selection have been observed so far in the present study. Therefore, we analyzed the gene expression of IGF-dependent intracellular signaling pathways in the pituitary gland and muscle (Mrf). Except for Tsc2 (*p* ≤ 0.01, Table 2), no significant effect of long-term selection on intracellular signaling was identified in the pituitary from sedentary DUhTP mice. By contrast, long-term selection in muscle tissue affected several DEGs involved in mTORC1 and mTORC2 pathways. Notably, the abundance of several mRNA transcripts coding for proteins mediating hormonal signals related to mTORC1 or mTORC2 activation or signaling was significantly suppressed (*p* ≤ 0.05) in sedentary DUhTP versus DUC mice, including Akt3, Gsk3b, Mtor, Clock, Pdpk1, Tsc1, Pten, Rictor, Rptor, Deptor, Rps6kp1 Foxo3, and Ei4einif1. Only the expression of Mlst8 as a part of the mTORC1/mTORC2 complex was increased in direct comparison and unremarkable after training. Notably, a known inhibitor of mTORC1 was significantly increased in DUhTP versus DUC mice (Akt1s1, *p* ≤ 0.001). In addition, mRNA expression of an inhibitor of protein translation (Eif4ebp1) and of Rps6 was significantly increased (*p* ≤ 0.001) in muscle of DUhTP compared to DUC mice.

In contrast to these hormone-sensitive signaling members, nutrient-sensitive mTORC1 signaling components were characterized by elevated mRNA expression in DUhTP versus DUC mice. Accordingly, mRNA expression of four components from the pentameric Ragulator complex (Lamtor1, 2, 4, and 5) was significantly increased in the muscle of sedentary DUhTP mice compared to controls (*p* ≤ 0.001; Table 3). In muscles of trained DUhTP mice, all members of the Lamtor family (Lamtor 1 to 5) were increased compared to trained DUC mice (*p* ≤ 0.05). Similarly, several members (sirtuin 2, 3, 4, 5, or 6) of the sirtuin family, a second nutrient-sensing protein family, were elevated in muscles from sedentary or trained DUhTP mice compared to their respective control group (*p* ≤ 0.05, Table 3). By contrast, mRNA expression encoding sirtuin1 was reduced in both DUhTP groups compared to DUC mice (*p* ≤ 0.001). In addition, four AMPK subunits were differentially regulated in both DUhTP groups compared to corresponding unselected control groups, respectively. Accordingly, mRNA expression of catalytic subunits Prkaa1 and -2 was reduced, whereas expression of Prkab1 and Prkag1 was elevated in the muscle of DUhTP mice (*p* < 0.05).

**Figure 2:**
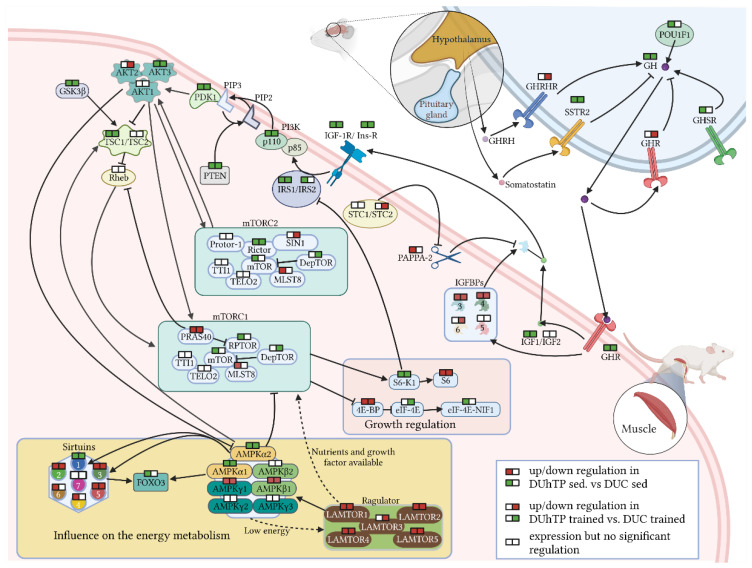
Gene expression of GH/IGF-related signaling cascades in the pituitary gland and muscle of phenotype-selected marathon mice (DUhTP) and unselected controls (DUC). The schematic summary presents results from Table 1, Table 2 and Table 3. The double boxes indicate regulation of mRNA expression when sedentary groups (left half of the box) or trained groups (right half of the box) were compared (red/green color: higher/lower gene expression in DUhTP versus DUC mice at *p* < 0.05; white boxes indicate no significant effects of genotype). Blunted arrows indicate inhibition, and dotted lines indicate indirect interactions between signaling compounds. Created on BioRender.com (accessed on 23/09/2021). Abbreviations are mentioned in Table 1, Table 2 and Table 3.

Results from Table 1, Table 2 and Table 3 are summarized in Figure 2. Additional effects of phenotypic selection and training on gene expression of growth factors or growth factor signaling are listed in Supplemental Table S1.

### 3.3. Effects of Phenotype Selection and Training on Intracellular Signal Transduction in the Muscle

**Figure 3:**
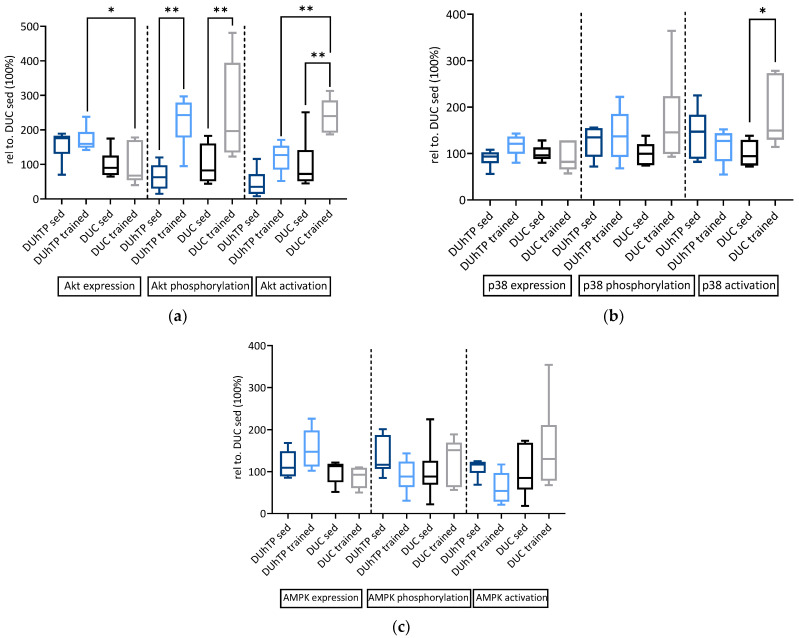
Effects of phenotype selection and endurance exercise on protein expression, phosphorylation, and specific activation of (**a**) AKT, (**b**) p38 MAPK, and (**c**) AMPK in *Musculus rectus femoris*. The analysis was performed by capillary immuno-electrophoresis (WES). Representative WES histograms created by Protein Simple Software are shown in Supplemental Figure S1. Data are presented as box plots and relative to sedentary DUC control mice, set to 100%. As an indicator of protein activation, the ratios were formed between protein phosphorylation and total protein expression. Statistical analysis was performed using one-way ANOVA. Significant differences are marked with asterisks (* *p* < 0.05, ** *p* < 0.01; *n* = 6). Abbreviations are defined in Table 1, Table 2 and Table 3.

**Figure 4:**
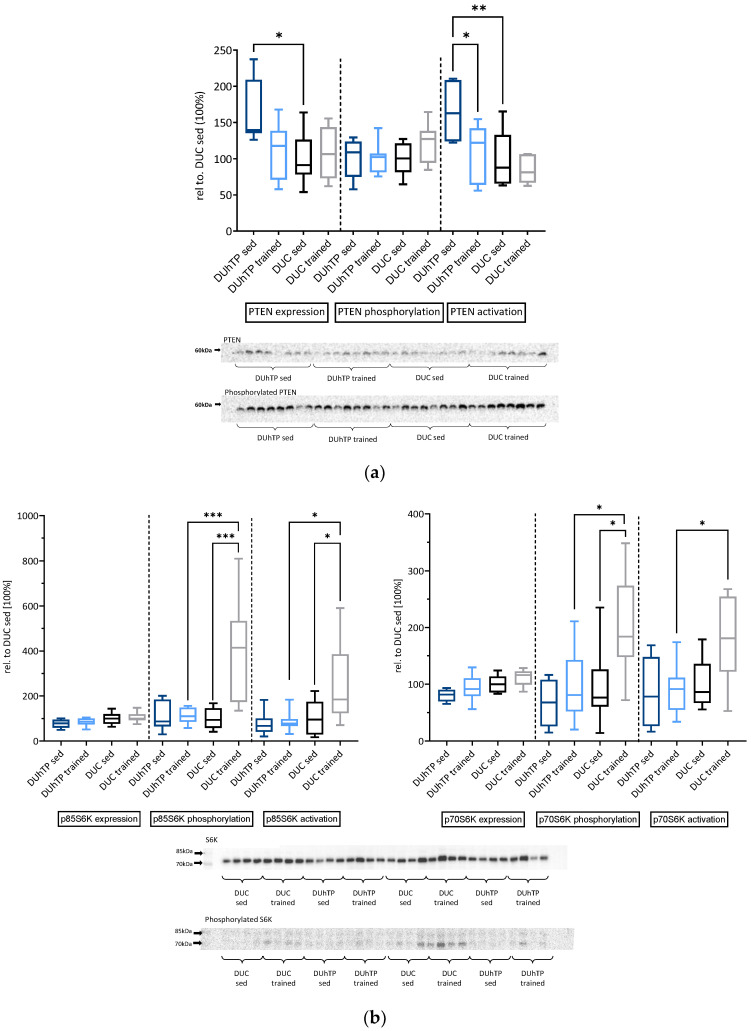
Effects of phenotype selection and endurance exercise on protein expression, phosphorylation, and specific activation of (**a**) PTEN and (**b**) p85S6K (left panel) and p70S6K (right panel) by Western immunoblot analysis in *Musculus rectus femoris*. The protein abundances in trained and untrained DUhTP (light/dark blue) and DUC mice (gray/black) were calculated relative to DUC sed (100%). Results are shown as box plots. Statistical analysis was performed using one-way ANOVA. Significant differences are marked with: * *p* < 0.05, ** *p* < 0.01, *** *p* < 0.001. Abbreviation: sed = sedentary.

In order to test the hypothesis of GH/IGF suppression in marathon mice, or whether reduced mRNA expression of signaling components also affects protein levels and activation, we performed a signal transduction study examining protein expression and phosphorylation in muscles from all four experimental groups. Expression of AKT protein was higher in muscles from trained DUhTP mice than trained DUC mice (*p* ≤ 0.05; Figure 3a). Training increased the absolute levels of phosphorylated AKT in both genetic groups compared to their sedentary control groups (*p* ≤ 0.05). Specific activation of AKT, however, was only observed in DUC but not in DUhTP mice (*p* ≤ 0.01). Accordingly, the specific activity was lower in muscles from DUhTP versus DUC mice (*p* ≤ 0.01). By contrast, the expression of PTEN was higher in sedentary DUhTP than in sedentary DUC mice (*p* ≤ 0.05; Figure 4a). Based on the higher ratios of unphosphorylated PTEN to phosphorylated PTEN, higher levels of active PTEN can be assumed in sedentary DUhTP mice compared to trained littermates (*p* ≤ 0.05) or sedentary controls (*p* ≤ 0.01). In addition, specific activation of p38 MAPK was found only in trained DUC (*p* ≤ 0.05; Figure 3b) but not in trained DUhTP mice. Expression and phosphorylation of AMPK were highly variable in different experimental groups (Figure 3c). Selection or training had no significant effect on AMPK expression or activation. In muscle extracts from trained DUC mice, higher levels of phosphorylated S6K were found compared to sedentary DUC littermates or trained DUhTP mice (*p* ≤ 0.001; Figure 4b). Also, the specific activity of S6K (ratio of phosphorylated to total protein) was higher in trained DUC mice than in both control groups (*p* ≤ 0.05).

## 4. Discussion

Preliminary work demonstrated that long-term selection for elevated endurance exercise capacities negatively affected body mass and muscle weight in male DUhTP marathon mice compared to unselected controls (Brenmoehl et al., submitted). Notably, exercise further reduced body mass and muscle weight in DUhTP mice but had no negative effect in unselected controls (DUC) (Brenmoehl et al., submitted). Therefore, we aimed to investigate the molecular basis of somatic and organ growth inhibition in DUhTP mice by comparing gene expression in the pituitary gland and femoral muscle tissue between phenotype-selected mice and unselected controls with and without previous training using RNAseq. This manuscript discusses endocrine signals from the pituitary gland for their potential effects on signal transduction in the muscle. The discussion is supported by an analysis of signal transduction on the protein level guided by predictions and models derived from RNA expression analysis.

### 4.1. Regulation of the Somatotropic Axis

In our non-inbred marathon mouse model DUhTP, characterized by superior running performance, expression of Pou1f1 was significantly reduced in pituitary glands, and notably, Pou1f1 expression was also suppressed by training in the pituitary gland of unselected control mice. These results may thus support the notion that reduced Pou1f1 expression in DUhTP, genetically fixed by several decades of phenotype selection, may be related to running performance in a physiological context. Pou1f1 represents a central pituitary transcription factor required for growth and development of the pituitary gland and expression of growth hormone (GH), thyrotropin, and prolactin (Prl) [11,12]. In fact, Gh expression was also suppressed in the pituitary gland of DUhTP mice, indicating physiological relevance of reduced Pou1f1 expression in DUhTP mice. Similar to Pou1f1, Ghsr was reduced in sedentary DUhTP versus DUC mice and with borderline significance also in trained versus sedentary DUC mice, suggesting not only reduced expression but also reduced secretion of GH from the pituitary gland in response to peripheral or metabolic signals [13]. Since serum levels of IGF1 were also reduced in sedentary DUhTP mice, central suppression of the GH/IGF axis can be assumed to result from phenotype selection. Reduced levels of IGF1 in DUhTP versus control mice were also found in a previous study [8], although the IGF1 concentrations described in the present study were on a lower level by factor 2. The reason for this discrepancy can be related to the high phenotypical variability of our model, which was described in detail before [8].

The GH/IGF-axis is centrally involved in growth and metabolism and regulated by physical activity. Therefore, it is tempting to speculate that exercise-related activation of GH expression in the pituitary gland, which is frequently described [14], would correlate with elevated circulating concentrations of IGF1, which might act as a potent mediator of GH-dependent muscle growth. However, a clear correlation of GH expression and elevated circulating IGF1 concentrations appears not to be present in response to exercise [5]. In his review, Frystyk discussed the discrepancy between pituitary GH-secretion, which is increased in response to exercise, and the lack of elevated circulating levels of free or bioactive IGF1 [5]. The lack of an exercise effect on the concentration of free IGF1 was confirmed more recently by a meta-analysis including 21 reports from the literature. However, this identified a positive effect of endurance and resistance exercise on absolute serum concentrations of IGF1 [15]. It was argued [5] that local production of IGF1 could be induced by GH-independent mechanisms, as suggested by Zanconato et al. [6]. In fact, in our study, local IGF1 was also elevated in the muscle of trained DUhTP mice compared to trained DUC mice, and muscle IGF1 thus contrasted and reduced GH mRNA expression in the pituitary gland of trained DUhTP mice.

However, the increases of muscle IGF1 on protein level were only weak and exclusively found in trained DUhTP mice, and we do not have evidence from our results that higher protein levels of IGF1 in muscle were functional for a number of reasons. First of all, in addition to the Ghr, several components from the PI3 signaling cascades were expressed at lower levels of mRNA, including Igf1, Igf1r, Insr, Irs1, Pten, Akt3, Gsk3b, Pdk, Tsc1, Rictor, and Rps6kb1 in both experimental DUhTP groups. By contrast, mRNA expression of Igfbp3 and Igfbp 4 was elevated in DUhTP mice. This finding clearly suggests coordinated inactivation of hormonal mTORC1 signaling, as discussed comprehensively by Philp et al. [16]. In this review, several studies collectively suggested adaptive muscle hypertrophy in the absence of IGF1R [7] or PI3K [17,18] signaling. While this evidence only supports the notion that hormone-dependent activation is not required for controlling muscle growth and metabolism in response to exercise, the present study identifies active downregulation of GH/IGF-related control of PI3K activation in the course of the selection experiment in DUhTP mice. The reductions of the GH/IGF system in the pituitary gland with coordinated downregulation of the PI3K in muscle may explain the reduced body and muscle mass in the DUhTP marathon mouse model (Brenmoehl et al., submitted). Collectively, downregulation of GH/IGF expression or signaling in DUhTP mice, characterized by the lower body and muscle mass than unselected controls, may suggest that body and muscle mass reductions may provide benefits for superior running performance. In fact, at least in a warm and humid environment, human runners with smaller body masses produced less heat than heavier runners and were able to run longer times and distances before a predefined rectal temperature was reached [19]. Since smaller runners are characterized by lower heat production, the authors stated that “smallness is an asset of distance running” [19]. Since marathon mice are characterized by massive fat cell browning and higher uncoupling protein 1 levels in fat tissues, including subcutaneous fat, correlating with elevated surface temperature [20], heat tolerance may be of critical importance to these mice. Accordingly, metabolic activity and heat tolerance could be related to central or peripheral somatotrophic growth inhibition in DUhTP marathon mice. The apparently successful management of heat stress in DUhTP mice, proven by the superior running capacities in this model, is highly relevant for heat stress management in farm animals and humans, which has never been more urgent than now given the increasing ambient temperatures during the climate crisis. Perhaps DUhTP mice can reveal novel mechanisms for heat stress defense in future studies.

### 4.2. Exercise-Related Activation of mTORC1 and mTORC2 in Muscle of DUC but Not of DUhTP Mice

In unselected control mice, training specifically induced activation of S6K without affecting systemic IGF1 concentrations or local IGF1 expression, which agrees with a recent report describing IGF1/AKT-independent activation of mTORC1 in response to resistance exercise in AKT1 knockout mice [21]. Accordingly, we also lack evidence for hormone-related activation of mTORC1 in DUC mice. In response to training, activation of mTORC1 in DUC mice fits with current concepts of exercise-related activation [22]. In fact, mTORC1 is considered a critical component for the muscle [23] since muscle growth during resistance training [24] depends on ribosomal biogenesis [25] and protein translation [4,26,27]. Clearly, and contrasting our findings in unselected controls, exercise-related activation of S6K was absent in DUhTP mice. The lack of mTORC1 activation in marathon mice could be related to multiple reductions of permissive signaling compounds described on the level of mRNA expression or to reduced serum IGF1 concentrations. In addition, the lack of mTORC1 activation could be due to the elevated expression of inhibitory proteins. Accordingly, the specific inhibitor of mTOR in the complex mTORC1, PRAS40 (gene Akt1s1), is upregulated in sedentary and trained DUhTP mice. This inhibitor is displaced by activated AKT [28], which is lacking in trained DUhTP mice. Since the negative effects of PRAS40 on S6K phosphorylation and Rheb-mediated mTORC1 activation can be blocked by insulin [29], and Insr and Irs1 were also repressed in muscle of DUhTP mice, we cannot rule out the notion that effects of insulin on mTORC1 could be toned down in muscle of DUhTP mice. Accordingly, multiple levels of mTORC1 repression composed of the interaction of activators and inhibitors and different hormonal systems can be considered in DUhTP mice. Importantly, the presence of multiple levels of mTORC1 repression can be related to the non-inbred background of DUhTP mice and speaks against a permissive function of mTORC1 for superior running performance in DUhTP mice. Notably, inhibition of mTORC1 by rapamycin improved mitochondrial function in a mouse model for myopathy [30].

Similar to mTORC1, we also have evidence that mTORC2 is activated in response to training in DUC but not in DUhTP mice since AKT was specifically activated in trained DUC mice compared to sedentary DUC mice or exercised DUhTP mice, and activation of AKT at serine 473 is a marker of mTORC2 activity [31]. Activation of mTORC2, in turn, is required for AKT/c-myc-dependent hypertrophic muscle growth in response to physical exercise [32]. However, we have no reason to postulate hormone-dependent activation of mTORC2 in DUC in response to exercise because local or systemic IGF1 concentrations were not increased in trained DUC mice. Accordingly, we have to assume, so far, unknown factors are involved in exercise-related mTORC2 activation. Notably, in sedentary DUhTP mice, elevated expression and higher levels of active PTEN, both in terms of total expression and reduced inactivation by protein phosphorylation of PTEN, could be related to the lack of mTORC2 activation in DUhTP mice in response to training. Inhibition of PTEN improved muscle function in Duchenne muscular dystrophy [33], and aerobic exercise had a negative effect on the expression of PTEN in mice [34]. Just recently, moderate training in rats was shown to block expression of PTEN, and it was discussed that thereby an age-related loss of muscle mass might be blocked on the level of the PI3K pathway [35,36]. Training also activated the p38 MAPK in the muscle of rats [36]. We identified activation of both PI3K and p38 MAPK in trained DUC mice but not in trained DUhTP mice, although we did not observe altered expression or activity of PTEN in response to exercise in our experimental system. While PI3K is thought to be related to hypertrophic growth and protein translation, as discussed earlier, p38 MAPK is a mediator of energy metabolic adaptation in response to exercise [37]. Accordingly, p38 MAPK can activate PGC1α on the protein level by direct interaction [38]. Furthermore, p38 MAPK can induce gene expression of PGC1α and GLUT4 by indirect mechanisms, e.g., via MEF2 on the level of mRNA expression [39,40].

Additional candidate genes were identified in the muscle of unselected control mice controlled by exercise (Clock, Ncam1, Fgfr4, and Hbegf). For these candidates, specific roles have been suggested with respect to metabolic adaptation [41], muscle innervation [42], training responses [43], or muscle cell differentiation [44]. It is possible that decades of selection under avoidance of inbreeding have enriched multiple mechanisms related to superior running performance in DUhTP mice, which might also warrant separate studies in the future.

### 4.3. Effects of Phenotype-Selection on mRNA Expression Related to Metabolic Cell Signaling

Neither exercise-related activation of signal transduction (AKT, S6K, and p38 MAPK) nor expression of Clock, Ncam1, or other candidate genes described in control mice were identified in the muscle of DUhTP mice. Accordingly, we must assume other pathways and mechanisms genetically fixed by long-term selection in the marathon mouse model. In fact, in muscle, the coordinated downregulation of GH/IGF-signaling was accompanied by substantially increased mRNA expression of pentameric Ragulator complex components, and except for Sirt1, also of several sirtuin family members. Notably, both protein families are regulated by signals related to energy metabolism but not by endocrine growth factors. The Ragulator complex is composed of Lamtor 1 to 5 and is required for leucine-dependent activation of mTORC1 [45]. Intriguingly, the Ragulator complex also activates AMPK, and therefore, has been identified as a molecular “switch between anabolism and catabolism” [46]. Under high energy conditions, the Ragulator complex activates anabolic mTORC1 signaling, whereas, under conditions of low energy availability, catabolic AMPK is activated [46]. In DUC mice, AMPK was not activated in response to training in contrast to mTORC1 and mTORC2. Since we observed abrogated anabolic signaling by coordinated reduction of hormone-induced signal transduction and a lack of exercise-induced activation of mTORC1 in the muscle of DUhTP mice, we might interpret massive induction of Ragulator complex expression in a context of AMPK-related metabolic control in the muscle of DUhTP mice. However, the coordinated induction of gene expression for the pentameric Ragulator complex in the muscle of DUhTP mice did not correlate with elevated phosphorylation of AMPK under the experimental conditions of the present study. Both catalytic subunits (Prkaa1/2) from the AMPK protein complex were reduced, whereas one beta and one gamma subunit were increased (Prkab1, Prkag1) in DUhTP mice. Effects on exercise tolerance [47], glucose uptake [47], glycogen content [48], mitochondrial mass [47], fat oxidation [49], and intracellular lipid content [50] have been described for the alpha, beta, and gamma subunits of the AMPK complex. Based on the differential control of AMPK subunits in DUhTP mice, we may assume multiple effects on metabolic control in their muscle. However, the classical concept of mutual anabolic versus metabolic control cannot be described or confirmed in marathon mice. In future experiments, the potential effect of elevated Ragulator complex expression on AMPK activation could be studied under more strict energy restriction conditions because the selection experiment was characterized by higher running intensities compared to the training units applied here.

From the strong effects on the expression of sirtuins in muscle, we may assume adaptive responses on the level of energy metabolism and protein acetylation. Sirtuins are a group of deacetylases and ADP-ribosylases with multiple effects on the level of DNA, RNA, protein, or metabolites in different cellular compartments [51]. In elderly men, resistance exercise training increased serum levels of Sirt1, 3, and 6 [52]. Since this increase was associated with elevated serum levels of telomerase and PGC-1α, beneficial effects of exercise were discussed in a context with sirtuins and PGC-1α [52]. In muscles from DUhTP mice, sirtuin 1 expression was abrogated, not directly supporting a joint effect of Sirt1 and 3 on mitochondrial biogenesis as discussed in our review [53]. Instead, multiple effects of Sirt2 to 6 may be assumed in the muscle of DUhTP mice in response to long-term phenotype selection characterized by multiple repeats of selection originating from a genetic outbred background.

This study has several limitations. First of all, we were unable to assess the effects of selection and training in both sexes. This is related to the fact that male mice were used when the phenotype selection experiment started decades ago. Nevertheless, the hypothesis should also be tested in females in future studies. Future studies will also examine in-depth metabolic control by the insulin receptor and glucose metabolism in our model.

## 5. Conclusions

To conclude, we have identified centrally reduced Pou1f1 and Gh mRNA expression in the pituitary gland of marathon mice, which correlated with reduced IGF1 serum concentrations in sedentary DUhTP mice. In muscle of DUhTP, but not in unselected control mice, coordinate downregulation of multiple components from the mTORC1 and -2 pathways was observed, whereas expression of IGFBPs was elevated in muscle. Downregulation of hormone-dependent signaling pathways in DUhTP mice, as demonstrated on the level of mRNA expression, coincided with abrogated activation of mTORC2 (AKT) and mTORC1 (S6K), which was well-observed in control mice in response to training. Accordingly, results on the level of protein appear to support results from pathway analysis on the level of mRNA expression. Therefore, we may conclude that central downregulation of the somatotropic axis and local downregulation of hormone-dependent mTORC activity represent adaptations as a response to long-term selection for high running performance in DUhTP mice. The downregulation of the somatotropic axis in DUhTP mice suggests not only that the somatotropic axis is not required for improved running performance but that it even needs to be suppressed for improved running performance in mice.

## Figures and Tables

**Table 1:** Effects of phenotype selection and exercise training on the GH/IGF system. List of comparative gene expression (Gene ID) as logarithmic fold change (log2FC) with corresponding false discovery rate (FDR) in the pituitary gland and skeletal muscle in the four comparison groups. Significant effects below a threshold of FDR ≤ 0.1 are marked in red (upregulated) or green (downregulated) and below an FDR ≤ 0.05 in bold.

Signaling Pathway		Comparison Parameters	Expression in the Pituitary Gland				Expression in Skeletal Muscle			
	Gene ID		DUhTP vs. DUC		DUC	DUhTP	DUhTP vs. DUC		DUC	DUhTP
			Sed	Trained	Trained vs. Sed		Sed	Trained	Trained vs. Sed	
GH axis	Pou1f1	log2FC	**−0.378**	−0.067	**−0.339**	−0.028				
		FDR	**0.002**	0.650	**0.027**	1.000				
	Gh	log2FC	−0.440	**−0.724**	0.378	0.095				
		FDR	0.066	**0.000**	0.158	1.000				
	Ghsr	log2FC	**−0.813**	−0.134	−0.683	−0.004				
		FDR	**0.016**	0.744	0.082	1.000				
	Sstr1	log2FC	0.676	−0.084	0.862	0.102				
		FDR	0.107	0.861	0.053	1.000				
	Sstr2	log2FC	**−1.367**	**−1.653**	−0.287	−0.573				
		FDR	**0.004**	**0.000**	0.668	1.000				
	Ghrhr	log2FC	−0.144	**0.493**	−0.176	0.462				
		FDR	0.545	**0.002**	0.427	0.087				
	Ghr	log2FC	−0.014	0.397	−0.190	0.221	**−0.291**	**−0.557**	−0.097	**−0.363**
		FDR	0.977	0.073	0.526	1.000	**0.034**	**0.000**	0.859	**0.025**
IGF system	Igf1	log2FC	−0.075	0.299	−0.563	−0.189	**−0.882**	**−1.722**	0.098	**−0.742**
		FDR	0.906	0.426	0.179	1.000	**0.000**	**0.000**	0.914	**0.002**
	Igf2	log2FC	**−4.277**	−0.280	**−3.217**	0.780	−0.332	−0.711	1.017	0.638
		FDR	**0.000**	0.843	**0.021**	1.000	0.569	0.138	0.303	0.363
	Igf1r	log2FC	−0.302	0.117	−0.209	0.211	**−0.480**	**−0.279**	−0.173	0.029
		FDR	0.069	0.487	0.267	0.989	**0.000**	**0.046**	0.670	0.913
	Igf2r	log2FC	−0.249	0.109	**−0.307**	0.050	**−0.717**	**−0.625**	0.013	0.105
		FDR	0.086	0.450	**0.049**	1.000	**0.000**	**0.000**	0.987	0.565
	Irs1	log2FC	0.164	0.394	−0.207	0.022	**−0.826**	**−1.161**	−0.021	−0.357
		FDR	0.579	0.054	0.445	1.000	**0.000**	**0.000**	0.985	0.091
	Irs2	log2FC	−0.334	−0.132	−0.068	0.134	**−1.119**	−0.331	−0.236	0.552
		FDR	0.271	0.648	0.868	1.000	**0.037**	0.562	0.920	0.474
	Insr	log2FC	−0.157	0.208	−0.204	0.161	**−0.604**	**−0.724**	−0.114	−0.235
		FDR	0.389	0.147	0.242	1.000	**0.000**	**0.000**	0.740	0.080
	Igfbp2	log2FC	**−1.494**	**−0.943**	−0.678	−0.127				
		FDR	**0.001**	**0.032**	0.220	1.000				
	Igfbp3	log2FC	−0.204	−0.106	−0.133	−0.035	0.364	**0.495**	−0.394	−0.263
		FDR	0.318	0.570	0.547	1.000	0.053	**0.006**	0.302	0.295
	Igfbp4	log2FC	0.368	−0.238	0.527	−0.079	0.211	**0.459**	0.066	**0.314**
		FDR	0.220	0.378	0.080	1.000	0.094	**0.000**	0.899	**0.030**
	Igfbp5	log2FC	−0.194	0.000	−0.175	0.019	−0.215	−0.107	−0.326	−0.218
		FDR	0.305	1.000	0.367	1.000	0.273	0.589	0.419	0.399
	Igfbp6	log2FC	−0.565	−0.525	−0.149	−0.109	0.174	**0.582**	−0.041	**0.367**
		FDR	0.395	0.344	0.859	1.000	0.289	**0.000**	0.955	**0.043**
	Igfbp7	log2FC	−0.153	**−0.577**	0.233	−0.191	0.179	**0.307**	0.015	0.142
		FDR	0.479	**0.000**	0.236	1.000	0.141	**0.006**	0.985	0.387
	Pappa2	log2FC	**−0.759**	**−0.566**	−0.090	0.103	−0.233	−0.341	0.560	0.453
		FDR	**0.000**	**0.006**	0.792	1.000	0.660	0.470	0.659	0.493
	Stc1	log2FC	−0.247	−0.003	−0.481	−0.237	−0.810	−0.627	−0.308	−0.124
		FDR	0.536	0.993	0.173	1.000	0.059	0.153	0.850	0.877
	Stc2	log2FC	0.511	0.667	0.289	0.445	0.550	**0.595**	0.240	0.285
		FDR	0.256	0.064	0.566	1.000	0.088	**0.049**	0.850	0.525
	Slc2a4	log2FC	0.178	−0.129	0.276	−0.031	**0.227**	**0.362**	0.134	**0.268**
		FDR	0.732	0.751	0.530	1.000	**0.033**	**0.000**	0.654	**0.033**

Abbreviations: GH: growth hormone; IGF: insulin-like growth factor; DUhTP: mouse line selected for high treadmill performance; DUC: unselected control mouse line; sed: sedentary; vs: versus; Pou1f1: POU class 1 homeobox 1; Ghsr: growth hormone secretagogue receptor; Sstr: somatostatin receptor; Ghrhr: growth-hormone-releasing hormone receptor; Ghr: growth hormone receptor; Igf1/2r: insulin-like growth factor receptor 1/2; Irs: insulin receptor substrate; Insr: insulin receptor; Igfbp: insulin-like growth factor binding protein; Pappa2: pappalysin-2; Stc: stanniocalcin; Slc2a4: glucose transporter type 4.

**Table 2:** Effects of phenotype selection and endurance exercise training on mRNA expression of components from hormone-dependent intracellular signaling cascades in the pituitary gland and muscle. The effects of selection or exercise are presented as logarithmic fold change (log2FC) with corresponding false discovery rate (FDR) in both tissues in four comparison groups. Significant effects below a threshold of FDR ≤ 0.1 are marked in red (upregulated) or green (downregulated) and below an FDR ≤ 0.05 in bold.

Functional Group		Comparison Parameters	Expression in the Pituitary Gland				Expression in Skeletal Muscle			
	Gene ID		DUhTP vs. DUC		DUC	DUhTP	DUhTP vs. DUC		DUC	DUhTP
			Sed	Trained	Trained vs. Sed		Sed	Trained	Trained vs. Sed	
Hormonal control of mTORC activity	Akt1	log2FC	−0.033	**−0.180**	0.088	−0.059	0.125	0.199	0.082	0.156
		FDR	0.834	**0.049**	0.476	1.000	0.368	0.116	0.877	0.384
	Akt2	log2FC	−0.113	−0.138	−0.016	−0.042	0.079	**0.254**	−0.028	0.146
		FDR	0.395	0.190	0.929	1.000	0.496	**0.010**	0.954	0.292
	Akt3	log2FC	−0.005	0.227	−0.070	0.162	**−0.668**	**−0.467**	−0.076	0.124
		FDR	0.983	0.060	0.695	1.000	**0.000**	**0.000**	0.881	0.508
	Gsk3b	log2FC	−0.239	0.002	−0.267	−0.026	**−0.684**	**−0.642**	−0.189	−0.147
		FDR	0.157	0.991	0.133	1.000	**0.000**	**0.000**	0.506	0.383
	Bmal1	log2FC	0.332	−0.064	−0.444	**−0.840**	−0.165	−0.650	−0.755	**−1.240**
		FDR	0.288	0.845	0.153	**0.012**	0.684	0.055	0.269	**0.001**
	Clock	log2FC	−0.093	0.153	−0.350	−0.105	**−0.539**	**−1.026**	**−0.509**	**−0.997**
		FDR	0.713	0.386	0.074	1.000	**0.000**	**0.000**	**0.012**	**0.000**
	Mtor	log2FC	−0.106	0.159	−0.207	0.058	**−0.330**	0.010	−0.142	0.198
		FDR	0.556	0.233	0.189	1.000	**0.005**	0.944	0.687	0.205
	Pik3ca	log2FC	−0.140	0.123	−0.203	0.060	**−0.532**	**−0.618**	−0.303	**−0.389**
		FDR	0.378	0.355	0.180	1.000	**0.000**	**0.000**	0.111	**0.003**
	Pik3cb	log2FC	−0.092	0.034	−0.086	0.041	**−0.400**	**−0.527**	−0.005	−0.133
		FDR	0.704	0.867	0.706	1.000	**0.011**	**0.000**	0.994	0.589
	Pik3c2a	log2FC	−0.131	0.156	−0.296	−0.009	**−0.986**	**−1.328**	−0.240	**−0.582**
		FDR	0.604	0.413	0.168	1.000	**0.000**	**0.000**	0.678	**0.020**
	Pdpk1	log2FC	−0.140	0.072	−0.163	0.049	**−0.797**	**−0.771**	−0.016	0.010
		FDR	0.508	0.700	0.413	1.000	**0.000**	**0.000**	0.980	0.968
	Tsc1	log2FC	0.019	**0.279**	−0.163	0.097	**−0.557**	**−0.447**	0.008	0.119
		FDR	0.946	**0.038**	0.352	1.000	**0.000**	**0.000**	0.991	0.441
	Tsc2	log2FC	**−0.254**	−0.046	−0.143	0.065	−0.182	0.047	0.048	**0.277**
		FDR	**0.008**	0.688	0.223	1.000	0.091	0.691	0.919	**0.025**
	Rheb	log2FC	−0.054	**−0.304**	0.198	−0.052	0.094	−0.028	0.061	−0.061
		FDR	0.710	**0.000**	0.077	1.000	0.388	0.805	0.881	0.701
	Pten	log2FC	−0.186	0.243	−0.361	0.069	**−0.678**	**−1.056**	0.017	**−0.361**
		FDR	0.404	0.166	0.083	1.000	**0.000**	**0.000**	0.981	**0.009**
	Rictor	log2FC	0.008	**0.347**	−0.227	0.112	**−0.550**	**−0.772**	−0.257	**−0.478**
		FDR	0.980	**0.015**	0.215	1.000	**0.004**	**0.000**	0.630	**0.044**
	Rptor	log2FC	−0.127	−0.011	−0.072	0.044	**−0.264**	−0.058	0.047	**0.252**
		FDR	0.283	0.932	0.585	1.000	**0.008**	0.599	0.919	**0.036**
	Deptor	log2FC	0.178	**0.582**	−0.122	0.281	−0.120	**−0.529**	0.150	−0.258
		FDR	0.576	**0.006**	0.709	1.000	0.501	**0.000**	0.783	0.217
	Rps6kb1	log2FC	−0.113	0.151	−0.184	0.080	**−0.341**	**−0.786**	−0.033	**−0.479**
		FDR	0.583	0.325	0.308	1.000	**0.013**	**0.000**	0.962	**0.002**
	Rps6	log2FC	−0.088	**−0.451**	**0.375**	0.011	**0.368**	**0.529**	0.048	0.209
		FDR	0.696	**0.001**	**0.034**	1.000	**0.000**	**0.000**	0.908	0.069
	Mlst8	log2FC	−0.002	−0.200	0.076	−0.122	**0.274**	0.169	0.104	−0.001
		FDR	0.994	0.090	0.645	1.000	**0.020**	0.154	0.808	0.996
	Mapkap1	log2FC	−0.008	−0.196	0.063	−0.126	0.095	0.155	0.074	0.135
		FDR	0.976	0.096	0.714	1.000	0.334	0.080	0.823	0.259
	Akt1s1	log2FC	−0.035	−0.225	0.232	0.042	**0.406**	**0.629**	−0.101	0.122
		FDR	0.900	0.145	0.215	1.000	**0.000**	**0.000**	0.813	0.472
	Telo2	log2FC	−0.230	**−0.352**	−0.009	−0.131	0.043	−0.054	0.088	−0.010
		FDR	0.085	**0.002**	0.971	1.000	0.805	0.738	0.883	0.973
	Eif4ebp1	log2FC	−0.194	−0.213	0.199	0.180	**0.563**	**0.862**	−0.048	0.251
		FDR	0.651	0.506	0.615	1.000	**0.000**	**0.000**	0.944	0.186
	Eif4enif1	log2FC	−0.004	0.086	−0.083	0.007	**−0.219**	−0.099	−0.065	0.056
		FDR	0.982	0.390	0.493	1.000	**0.021**	0.318	0.866	0.722
	Foxo3	log2FC	−0.018	0.194	−0.142	0.070	**−0.388**	0.209	−0.015	**0.582**
		FDR	0.952	0.195	0.460	1.000	**0.002**	0.093	0.987	**0.000**

Abbreviations: DUhTP: mouse line selected for high treadmill performance; DUC: unselected control mouse line; sed: sedentary; vs: versus; Akt: protein kinase B; Gsk3b: glycogen synthase kinase 3 beta; Bmal1: brain and muscle ARNT-like 1; Clock: circadian locomotor output cycles kaput; Mtor: mechanistic target of rapamycin; Pik3c: phosphatidylinositol 3-kinase subunits; Pdpk1: phosphoinositide-dependent kinase-1; Tsc: tuberous sclerosis complex; Rheb: Ras homolog enriched in brain; Pten: phosphatase and tensin homolog; Rictor: rapamycin-insensitive companion of mammalian target of rapamycin; Rptor: regulatory-associated protein of mTOR; Deptor: DEP domain-containing mTOR-interacting protein; Rps6kb1: ribosomal protein S6 kinase beta-1; Rps6: ribosomal protein S6; Mlst8: target of rapamycin complex subunit LST8; Mapkap1: target of rapamycin complex 2 subunit; Akt1s1: proline-rich AKT1 substrate 1; Telo2: telomere length regulation protein TEL2 homolog; Eif4ebp1: eukaryotic translation initiation factor 4E-binding protein 1; Eif4enif1: eukaryotic translation initiation factor 4E transporter; Foxo3: forkhead box O3.

**Table 3:** Effects of phenotype selection and endurance exercise on mRNA expression coding for proteins and protein-complex subunits related to metabolic cell signaling in the pituitary gland and muscle. The effects of selection and exercise are presented as logarithmic fold change (log2FC) with corresponding false discovery rate (FDR) in the pituitary gland and skeletal muscle in four comparison groups. Significant regulations below a threshold of FDR ≤ 0.1 are marked in red (upregulated) or green (downregulated) and below an FDR ≤ 0.05 in bold.

Signaling Pathway Members		Comparison Parameters	Expression in the Pituitary Gland				Expression in Skeletal Muscle			
	Gene ID		DUhTP vs. DUC		DUC	DUhTP	DUhTP vs. DUC		DUC	DUhTP
			Sed	Trained	Trained vs. Sed		Sed	Trained	Trained vs. Sed	
Lamtors	Lamtor1	log2FC	0.035	**−0.265**	0.237	−0.063	**0.470**	**0.525**	0.131	0.186
		FDR	0.887	**0.049**	0.159	1.000	**0.000**	**0.000**	0.593	0.109
	Lamtor2	log2FC	−0.097	**−0.640**	**0.427**	−0.116	**0.377**	**0.331**	−0.029	−0.076
		FDR	0.710	**0.000**	**0.033**	1.000	**0.000**	**0.000**	0.951	0.611
	Lamtor3	log2FC	0.086	−0.049	0.022	−0.112	0.191	**0.238**	−0.077	−0.030
		FDR	0.571	0.708	0.904	1.000	0.068	**0.017**	0.850	0.868
	Lamtor4	log2FC	0.090	**−0.395**	0.416	−0.069	**0.490**	**0.486**	0.014	0.010
		FDR	0.751	**0.021**	0.050	1.000	**0.000**	**0.000**	0.983	0.959
	Lamtor5	log2FC	0.019	−0.097	0.199	0.084	**0.446**	**0.399**	0.069	0.023
		FDR	0.955	0.609	0.329	1.000	**0.000**	**0.000**	0.876	0.905
Sirtuins	Sirt1	log2FC	−0.247	0.060	−0.258	0.049	**−0.554**	**−0.899**	0.040	−0.305
		FDR	0.216	0.771	0.207	1.000	**0.000**	**0.000**	0.956	0.128
	Sirt2	log2FC	0.021	0.002	0.043	0.024	**0.260**	**0.384**	0.011	0.134
		FDR	0.896	0.988	0.739	1.000	**0.002**	**0.000**	0.986	0.249
	Sirt3	log2FC	0.205	0.074	0.166	0.034	**0.480**	**0.454**	0.106	0.080
		FDR	0.105	0.567	0.228	1.000	**0.000**	**0.000**	0.839	0.718
	Sirt4	log2FC	−0.098	−0.028	−0.025	0.045	**0.297**	0.134	0.191	0.029
		FDR	0.567	0.858	0.904	1.000	**0.028**	0.333	0.591	0.905
	Sirt5	log2FC	0.064	0.239	0.044	0.219	**0.570**	**1.177**	−0.093	**0.514**
		FDR	0.836	0.196	0.884	1.000	**0.001**	**0.000**	0.909	**0.012**
	Sirt6	log2FC	0.041	−0.136	0.084	−0.092	**0.337**	0.285	0.035	−0.017
		FDR	0.876	0.395	0.692	1.000	**0.041**	0.078	0.968	0.959
	Sirt7	log2FC	−0.227	**−0.339**	0.097	−0.016	−0.047	0.104	0.025	0.177
		FDR	0.090	**0.002**	0.549	1.000	0.810	0.541	0.978	0.437
AMPK subunits	Prkaa1	log2FC	−0.046	0.133	−0.202	−0.024	**−0.680**	**−0.844**	−0.105	−0.269
		FDR	0.842	0.364	0.224	1.000	**0.000**	**0.000**	0.818	0.099
	Prkaa2	log2FC	0.051	**0.351**	−0.203	0.096	**−0.672**	**−0.885**	−0.084	−0.297
		FDR	0.872	**0.046**	0.372	1.000	**0.000**	**0.000**	0.881	0.080
	Prkab1	log2FC	0.066	0.028	0.083	0.045	**0.355**	**0.315**	−0.037	−0.077
		FDR	0.720	0.852	0.610	1.000	**0.010**	**0.021**	0.957	0.736
	Prkab2	log2FC	0.077	0.277	−0.121	0.079	−0.111	−0.101	0.031	0.041
		FDR	0.762	0.077	0.575	1.000	0.577	0.592	0.974	0.897
	Prkag1	log2FC	−0.091	**−0.286**	0.186	−0.009	**0.437**	**0.527**	0.026	0.115
		FDR	0.569	**0.007**	0.177	1.000	**0.000**	**0.000**	0.960	0.434
	Prkag2	log2FC	0.029	0.099	−0.186	−0.115	0.244	0.217	0.077	0.049
		FDR	0.883	0.405	0.167	1.000	0.150	0.188	0.917	0.864
	Prkag3	log2FC					0.297	−0.043	0.330	−0.010
		FDR					0.163	0.856	0.498	0.981

Abbreviations: DUhTP: mouse line selected for high treadmill performance; DUC: unselected control mouse line; sed: sedentary; vs: versus; mTORC1: mechanistic target of rapamycin complex 1; Lamtor: Ragulator-Rag complex; Sirt: sirtuin; AMPK: 5′-AMP-activated protein kinase; Prka: 5′-AMP-activated protein kinase subunits.

## Data Availability

All data are available in the present manuscript, in the Supplement, in referenced manuscripts [9], or upon the request from the corresponding author.

## Supplementary Materials

The following are available online at https://www.mdpi.com/article/10.3390/cells10123418/s1, Table S1: Effects of phenotype selection and endurance exercise on mRNA expression of other growth factors and receptors in the pituitary gland and muscle. Figure S1: Capillary immuno-electrophoresis (WES) spectra.
