# Supplementary material for: Central Suppression of the GH/IGF Axis and Abrogation of Exercise-Related mTORC1/2 Activation in the Muscle of Phenotype-Selected Male Marathon Mice (DUhTP)
Source: Cells. 2021 Dec 4;10(12):3418. doi: 10.3390/cells10123418 (PMC8699648; doi:10.3390/cells10123418)
Supplement: Supplementary file 1 [file cells-10-03418-s001.zip › cells-1480085-supplementary.pdf]

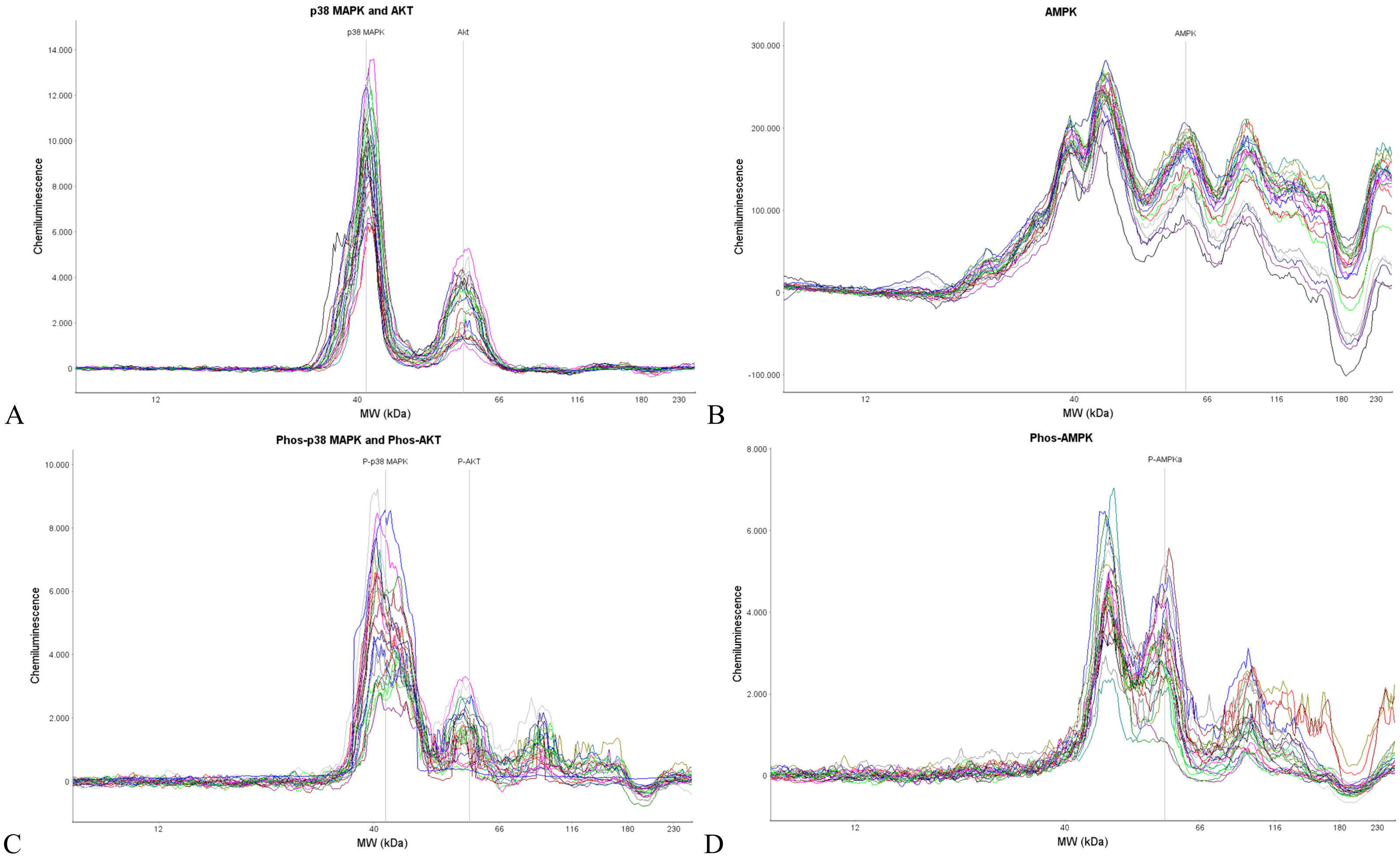

Supplement Figure S1 Effects of phenotype selection and endurance exercise on protein expression and phosphorylation (Phos) of AKT and p38 MAPK (A and C), as well as AMPK (B and D) in Musculus rectus femoris. The analysis was performed by capillary immuno-electrophoresis (WES). The WES spectra were created by Protein Simple Software Compass and show the chemiluminescence for every single capillary. Analysis and peak finding was performed in the software Compass with the following settings for peak finding: For AKT and p38 (phosphorylated and total): Threshold 10.0, Width 9.0, area calculation by Gaussian Fit; for AMPK (phosphorylated and total): Threshold 10.0, Width 6.0, area calculation by Dropped Lines. The results of spectra evaluations are shown in Figure 3. Abbreviations are mentioned in Tables 1-3.

Supplemental Table S1. Effects of phenotype selection and endurance exercise on mRNA expression of other growth factors and receptors in the pituitary gland and muscle. The selection and exercise-mediated effects are presented as logarithmic fold change (log2FC) with corresponding false discovery rate (FDR) in the pituitary gland (left) and skeletal muscle (right) in four comparison groups. Significant regulations below a threshold of FDR ≤ 0.1 are marked in red (upregulated) or green (downregulated) and below an FDR ≤ 0.05 in bold.

Signaling pathway members	Gene ID	Comparison parameters	Expression in the pituitary gland				Expression in skeletal muscle			
			DUhTP vs DUC		DUC	DUhTP	DUhTP vs DUC		DUC	DUhTP
			sed	trained	trained vs sed		sed	trained	trained vs sed	
Growth factors and associated genes	Gap43	log2FC	**0.856**	0.232	0.637	0.013				
		FDR	**0.017**	0.553	0.134	1.000				
	Sp1	log2FC	-0.074	0.221	-0.185	0.110	**-0.448**	**-0.575**	-0.225	**-0.352**
		FDR	0.733	0.112	0.283	1.000	**0.000**	**0.000**	0.429	**0.021**
	Atg13	log2FC	-0.113	-0.130	0.018	0.001	**-0.257**	-0.172	0.012	0.097
		FDR	0.482	0.305	0.931	1.000	**0.011**	0.089	0.986	0.523
	Eif4e3	log2FC	-0.260	-0.151	-0.113	-0.004	-0.005	**-0.250**	0.148	-0.097
		FDR	0.069	0.270	0.514	1.000	0.976	**0.041**	0.691	0.616
	Foxo1	log2FC	0.090	0.134	-0.098	-0.054	-0.339	**0.602**	0.000	**0.941**
		FDR	0.719	0.450	0.673	1.000	0.223	**0.017**	1.000	**0.001**
	Ulk1	log2FC	-0.068	-0.003	-0.062	0.003	**-0.230**	-0.021	-0.101	0.107
		FDR	0.724	0.987	0.733	1.000	**0.044**	0.872	0.801	0.524
	Il18	log2FC	0.336	**0.357**	-0.042	-0.021	0.403	0.293	0.083	-0.027
		FDR	0.095	**0.044**	0.890	1.000	0.320	0.460	0.968	0.971
	Il18r1	log2FC	**1.641**	**1.492**	-0.035	-0.184				
		FDR	**0.000**	**0.000**	0.973	1.000				
	Cxcl1	log2FC	**-2.160**	**-1.971**	0.413	0.602				
		FDR	**0.011**	**0.010**	0.708	1.000				
	Cxcl13	log2FC	**-1.062**	**-0.894**	-0.111	0.058	-0.155	-0.082	0.888	0.960
		FDR	**0.010**	**0.023**	0.863	1.000	0.820	0.888	0.461	0.154
	Cxcl14	log2FC	-0.249	**-0.488**	0.086	-0.154	0.289	0.224	0.218	0.153
		FDR	0.207	**0.002**	0.725	1.000	0.086	0.177	0.627	0.530
	Nrtn	log2FC	0.212	**-0.923**	**0.929**	-0.206	**0.785**	0.436	0.278	-0.071
		FDR	0.728	**0.012**	**0.043**	1.000	**0.013**	0.168	0.819	0.897
	Ret	log2FC	**-0.756**	**-1.004**	0.378	0.130	**-0.610**	-0.215	0.010	0.405
		FDR	**0.016**	**0.000**	0.317	1.000	**0.001**	0.287	0.993	0.104
	Ncam1	log2FC	-0.103	**0.463**	-0.175	**0.392**	-0.564	**-1.360**	**1.307**	0.511
		FDR	0.580	**0.000**	0.284	**0.030**	0.121	**0.000**	**0.006**	0.280
	Muc15	log2FC	**2.720**	**2.788**	-0.400	-0.333				
		FDR	**0.000**	**0.000**	0.720	1.000				
	Npy	log2FC	**2.440**	0.779	**2.212**	0.551				
		FDR	**0.011**	0.401	**0.050**	1.000				

	Stat5a	log2FC	-0.173	-0.566	-0.003	-0.396	0.342	0.337	-0.180	-0.185
		FDR	0.480	0.001	0.994	0.353	0.019	0.019	0.683	0.365
	Fgf15	log2FC	-0.767	1.439	-0.629	1.577				
		FDR	0.351	0.014	0.444	0.161				
	Fgf22	log2FC	0.863	0.650	-0.004	-0.218				
		FDR	0.038	0.107	0.995	1.000				
	Fgf23	log2FC	1.161	1.238	0.012	0.088				
		FDR	0.070	0.028	0.993	1.000				
	Fgf13	log2FC	-0.069	0.238	0.020	0.327	-0.182	-0.502	0.118	-0.202
		FDR	0.892	0.441	0.972	1.000	0.092	0.000	0.717	0.136
	Fgf20	log2FC	0.442	-0.661	0.544	-0.560	0.652	1.107	-0.769	-0.314
		FDR	0.481	0.173	0.347	1.000	0.146	0.012	0.488	0.632
	Fgf6	log2FC					-0.384	-0.446	0.044	-0.018
		FDR					0.086	0.036	0.969	0.970
	Fgfbp1	log2FC	-2.349	-0.049	-2.033	0.267	2.175	2.751	-0.892	-0.316
		FDR	0.018	0.969	0.075	1.000	0.001	0.000	0.643	0.759
	Fgfr2	log2FC	0.145	0.316	-0.028	0.143	0.177	0.842	-0.380	0.285
		FDR	0.713	0.228	0.951	1.000	0.709	0.032	0.800	0.628
	Fgfr4	log2FC	1.241	0.559	0.965	0.283	1.001	-0.228	1.097	-0.132
		FDR	0.004	0.179	0.070	1.000	0.001	0.481	0.012	0.800
	Fgfrl1	log2FC	0.416	-0.019	0.346	-0.089	0.437	0.341	0.042	-0.053
		FDR	0.040	0.941	0.133	1.000	0.002	0.015	0.953	0.832
	Fgfr1op2	log2FC	-0.194	-0.041	-0.070	0.083	-0.242	-0.410	-0.123	-0.291
		FDR	0.043	0.701	0.565	1.000	0.028	0.000	0.718	0.026
	Egfr	log2FC	-0.035	0.407	-0.363	0.080	-0.548	-0.271	-0.041	0.236
		FDR	0.914	0.013	0.080	1.000	0.002	0.129	0.962	0.342
	Egflam	log2FC	0.128	0.713	-0.468	0.116	0.419	0.424	0.141	0.146
		FDR	0.625	0.000	0.033	1.000	0.002	0.001	0.761	0.464
	Megf10	log2FC	-1.463	-1.023	-0.585	-0.145	-0.526	-1.918	0.731	-0.661
		FDR	0.000	0.000	0.123	1.000	0.285	0.000	0.488	0.304
	Hbegf	log2FC	-0.206	-0.727	0.423	-0.097	0.350	-0.041	0.956	0.566
		FDR	0.624	0.008	0.220	1.000	0.091	0.859	0.000	0.014
Estrogen receptors	Esr1	log2FC	-0.317	0.190	-0.454	0.053	-0.175	-0.080	-0.138	-0.043
		FDR	0.022	0.159	0.008	1.000	0.279	0.628	0.794	0.875
	Esr2	log2FC	-1.295	-2.302	0.657	-0.351				
		FDR	0.096	0.000	0.368	1.000				

Abbreviations: DUhTP: mouse line selected for high treadmill performance; DUC: unselected control mouse line; sed: sedentary; vs: versus; Gap43: Growth associated protein; Sp1: Transcription factor Sp1; Atg13: Autophagy-related protein; Eif4e3: Eukaryotic translation initiation factor 4e-3; Foxo1: Forkhead box protein O1; Ulk1: Serin/threonine-protein kinase ULK1; Il18: Interleukin-18; Il18r1: Il18 receptor 1; Cxcl: C-X-C motif chemokine; Nrtn: Neurturin; Ret: Proto-oncogene tyrosine-protein kinase receptor Ret; Ncam1: Neural cell adhesion molecule 1; Muc15: Mucin-15; Npy: Neuropeptide Y; Stat5a: Signal transducer and activator of transcription 5a; Fgf:

Fibroblast growth factor; Fgfbp: Fgf binding protein; Fgfr: Fgf receptor; Fgfrl: Fgf receptor-like; Fgfr1op2: Fgfr1 oncogene partner 2; Egfr: Epidermal growth factor receptor; Egflam: Pikachurin; Megf10: Multiple epidermal growth factor-like domains protein 10; Hbegf: Heparin-binding Egf-like growth factor; Esr: estrogen receptor
